# Stress Concentration and Mechanical Strength of Cubic Lattice Architectures

**DOI:** 10.3390/ma11071146

**Published:** 2018-07-05

**Authors:** Paul Lohmuller, Julien Favre, Boris Piotrowski, Samuel Kenzari, Pascal Laheurte

**Affiliations:** 1 Laboratoire d’Etude des Microstructures et de Mécanique des Matériaux LEM3 UMR CNRS 7239, Arts et Métiers ParisTech Campus de Metz, Université de Lorraine, F-57078 Metz, France; boris.piotrowski@ensam.eu (B.P.); pascal.laheurte@univ-lorraine.fr (P.L.); 2 Institut Jean Lamour, UMR 7198 CNRS-Université de Lorraine, Campus Artem, F-54011 Nancy, France; samuel.kenzari@univ-lorraine.fr; 3 Laboratory of Excellence on Design of Alloy Metals for Low-mAss Structures (DAMAS), Université de Lorraine, F-54011 Nancy, France; 4 Mines Saint-Etienne, Univ Lyon, CNRS, UMR 5307 LGF, Centre SMS, Departement PMM, F-42023 Saint-Etienne, France; julien.favre@emse.fr

**Keywords:** lattice structures, porous materials, 3D surface maps, finite element, fatigue, plasticity

## Abstract

The continuous design of cubic lattice architecture materials provides a wide range of mechanical properties. It makes possible to control the stress magnitude and the local maxima in the structure. This study reveals some architectures specifically designed to reach a good compromise between mass reduction and mechanical strength. Decreased local stress concentration prevents the early occurrence of localized plasticity or damage, and promotes the fatigue resistance. The high performance of cubic architectures is reported extensively, and structures with the best damage resistance are identified. The fatigue resistance and S–N curves (stress magnitude versus lifetime curves) can be estimated successfully, based on the investigation of the stress concentration. The output data are represented in two-dimensional (2D) color maps to help mechanical engineers in selecting the suitable architecture with the desired stress concentration factor, and eventually with the correct fatigue lifetime.

## 1. Introduction

Recent progress in manufacturing technologies based on additive manufacturing [1,2] and welded assemblies [3] open new possibilities for the production of architectured materials with open porosities. Lattice periodic structures illustrate the appearance of new hybrid materials [4] combining high specific strength with ultra-low specific density, usually out of reach for any bulk materials. These porous structures combine properties that are usually considered as incompatible, and can be positioned in the blank areas on the strength/density material selection chart [3,5].

As a result of the promising specific properties of these materials, significant efforts have been made to highlight the importance of choosing the optimal architecture offering the best specific mechanical strength. The research procedure is usually iterated by trial and error; small alterations are operated on a well-known structure, and the benefits of each design are presented one by one. For instance, the well-known octet-truss [6,7] has been edited to make a new structure called an IsoTruss [8], with a superior specific stiffness and improved isotropy. To accelerate the optimization procedure, new design rules have emerged. The use of Bravais lattices provides simple rules to produce periodic structures [9]. Some recent works have shown that it is possible to use crystallography formalism to design very versatile complex architectures by just using a point group and two architectural parameters [10]. It was possible to define a large range of structures with variable stiffness and Poisson ratio. New solutions for the mass reduction have emerged from this formalism by developing structures with a high specific stiffness. The current work proposes an investigation of the stress concentration in a wide range of topologies. It provides an indication on the plasticity onset and thus on the associated fatigue resistance.

The specific stiffness of the lattice structures varies with the square of the specific density [10,11]. However, it was shown that the power exponent could deviate significantly from its nominal value of two, depending on the architecture [10]. Likewise, the yield strength and fatigue strength are expected to follow a power law of density. According to the early works of Ashby et al. [11], the power law coefficient is expected to be three/two for the dependence of yield stress on the specific density for a stochastic foam. Here again, some discrepancies on the power exponent are expected because of the effect of architecture. Some architectures could be more prone to stress concentration, leading to an early onset of plasticity and damage. Therefore, any architecture minimizing the stress concentration with a low density would be of high interest for mechanical engineers. This work aims to identify these optimal structures by evaluating the stress concentration coefficient as a function of density. Once the stress concentration is known, it is possible to discuss the onset of plasticity and damage. By transposing the data from the bulk materials into the case of the porous architectures, it becomes possible to give a first estimation of the possible fatigue lifetime. The ultimate goal of this work is to provide guidelines for mechanical engineers on ways to save mass and material consumption by producing architectured materials from known bulk materials, while preserving high mechanical strength and a controlled lifetime.

The second section will explain the architecture generation procedure and the associated numerical simulations. The third section details the determination of the stress concentration factor to discuss the heterogeneities of the stress field. The fourth part focuses on the application for mechanical design, and on the capability to predict the fatigue lifetime from the stress concentration factors.

## 2. Materials and Methods

**Figure 1:**
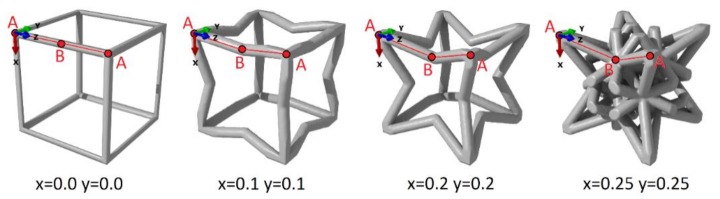
Cubic lattices designed by setting the (x, y) coordinates of the B point.

The cubic lattice structures are generated using crystallographic rules and symmetry operations. A cluster of points defines the architecture, and the struts correspond to the shortest possible segments between the nodes. This definition of struts is consistent with the concept of chemical bonding in crystals. The lattice design is defined by a set of two initial points belonging to the lattice network. Point A is set at the origin, and the position of point B is set by two parameters, x and y. Figure 1 illustrates some structures defined by points A and B. The structures are represented with a strut radius equal to 0.05 times the lattice parameter. The position along the z-axis must be set to 0.5 times the lattice parameter to produce a continuous network. Then the use of symmetry operations of the $m\bar{3}m$ space group on points A and B results in an infinite network of points corresponding to the possible positions of atoms in a crystal. The whole procedure is not described in this study, but interested readers should turn to the work of Favre et al. [10]. The case x = 0.0 and y = 0.0 results in a primitive cubic lattice (Figure 1), and the progressive increase of both x and y forms an auxetic re-entrant structure, called a hexatruss [12,13]. The further increase results in complex structures with a high struts density (for example, x = 0.25 y = 0.25 in Figure 1), and finally reaches the upper bound x = 0.5 and y = 0.5, corresponding to the centered cubic lattice. This technique provides a continuum between a known cubic Bravais lattice, such as primitive, face-centered, or cube-centered, and new complex architectures. 

The one-dimensional (1D) struts lattice network is converted into a three-dimensional (3D) object with struts, with a circular section. The radius of the struts is given as a fraction of the lattice parameter. For instance, if the lattice parameter is 1 mm, then the radius would be a fraction of 1 mm, and the computed stress unit will be in megapascals. The generation of 3D structures is operated using Openscad (version 2015.03-2) and Autodesk Meshmixer software (version 3.2.37). The 3D object is exported in ‘.stl’ format, and then it is post-processed by Tetgen, a meshing tool that is used to produce a model suitable for finite elements analysis. The 3D model is imported to Abaqus v6.13. The deformation applied on the lattice is a small compressive strain of ε = 1%. The modeling technique is further detailed by Favre et al. [10]. The opposite facets of the representative volume are symmetry planes, corresponding to a frictionless sliding. The material has homogeneous isotropic elastic properties. Simulations were conducted on an elastic material with a Young’s modulus E = 1 GPa, representative of the ABS polymer (Acrylonitrile Butadiene Styrene) that is used in commercial 3D printers. The output data were post-treated by Python scripts to determine the volume and the stress of each element. The statistical analysis of the stress distribution over the population of the elements provides some reliable values of the maximal stress, denoted $\sigma_{m}$, and of the average stress, denoted $\bar{\sigma }$. Furthermore, the macroscopic compression stress, denoted $\tilde{\sigma }$, is computed from the sum of all of the resulting forces applied on the outer surface of the cube bearing the external compressive pressure. 

During the elastic compression of the lattice structures, the macroscopic stress applied on the outer surface of the cube $\tilde{\sigma }$ is proportional to the macroscopic strain, and the proportionality coefficient is the apparent Young’s modulus E, reported by Favre et al. [10]. The lattice is considered as a homogeneous equivalent material, and the mechanical design relies on the value of $\tilde{\sigma }$, applied on the representative elementary volume. The stress concentration is defined by the ratio of the maximal stress to the macroscopic stress $\tilde{\sigma }$. It becomes a useful information for mechanical engineers, as it connects the effective macroscopic stress to the microscopic stress localizations.

Thereby, the stress concentration factor K is defined as the ratio between the maximal stress $\sigma_{m}$ and the external stress $\tilde{\sigma }$ applied on the lattice, as follows: $K=\sigma_{m}/\tilde{\sigma }$. This factor provides an estimation of the localized stress maximum. A second stress concentration factor $\bar{K}$ is computed by the ratio of the average stress in the lattice to the external stress: $\bar{K}=\bar{\sigma }/\tilde{\sigma }$. This factor illustrates the average stress concentration over the whole volume of the lattice, regardless of the localized stress maximum.

## 3. Result

### 3.1. Stress Field and Maximum

**Figure 2:**
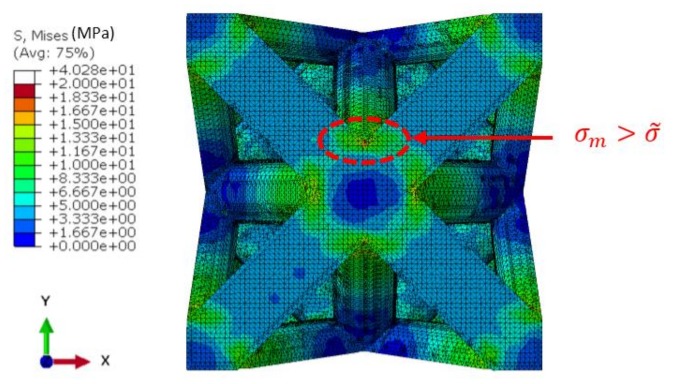
Von Mises stress field in an FCC lattice (x = 0.0 and y = 0.5), and stress concentration at nodes.

The finite element model provides a large set of information on the heterogeneous stress field in the lattices. Figure 2 illustrates the stress field in the case of an octet-truss lattice (FCC, x = 0.0 and y = 0.5). The stress concentration is the highest at the outer surface of the intersections of the beams. The magnitude of the Von Mises stress is about ten times larger than in the median part of the beams. This stress concentration at the surface is likely to result in localized plasticity and crack initiation.

**Figure 3:**
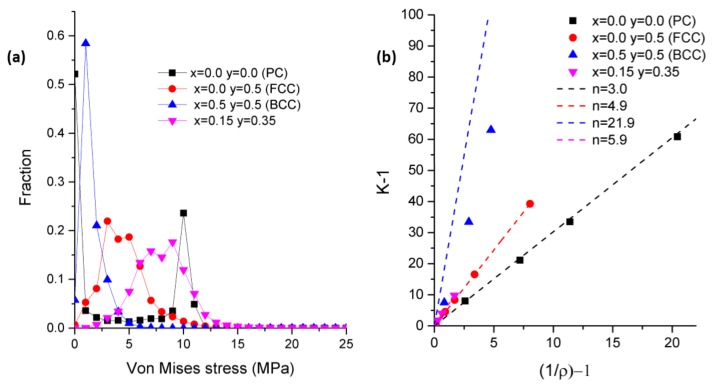
(**a**) Von Mises stress distribution in the set of elements for different architecture parameters; (**b**) stress concentration factor K as a function of density *ρ*.

Unfortunately, the analysis of the maximum stress is not straightforward, and it is preferable to avoid a simplistic search of the maximal stress value. Indeed, the finite element method often results in singularities in the stress field because of the possible distortion of some elements. Consequently, the evaluation of the maximal stress cannot rely on a single element, and a statistical approach must be set. The stress was analyzed over the complete population of the elements, and the distribution as a function of the volume fraction was plotted in Figure 3a. Depending on the architecture, the distribution exhibits a series of peaks for the low stress (<20 MPa for ε = 1% and E = 1 GPa). For the larger stress values, the fraction tends to zero, and the stress value is not representative of the effective stress field. For such distributions, taking the maximal value would result in poor accuracy. Therefore, the maximal stress $\sigma_{m}$ was defined as the mean value of 1% of the elements having the largest stress value. In other words, the maximal stress is the mean value within the tail of the distribution.

The factor K varies as a power law of density [11], as follows:

(1) $$K\;=\;1/\rho^{m}\;=\;\rho^{-m}$$

However, Equation (1) does not represent properly the variation of K with density. Instead, K follows a linear relation with 1/*ρ*. Then, the variation of K is represented by the following:

(2) $$K=n_{1}\times \frac{1}{\rho }+n_{2}\;$$

with *n*_1_ and *n*_2_ being two parameters depending on the architecture. For *ρ* = 1, there is naturally no stress concentration, and K = 1. Therefore, Equation (2) becomes the following:

(3) $$K=1+n\times \left(\frac{1}{\rho }-1\;\right)$$

with *n* a parameter to be determined. Figure 3b illustrates K  − 1 as a function of 1/*ρ* − 1, and the relation is linear with a slope equal to *n*. The fit of K values using the Equation (3) yields to a correlation coefficient *r*^2^ above 0.98.

The coefficient denoted n has been determined for every structure in the x–y parametric plan. The same relationship holds for the global stress concentration factor $\bar{K}$. It results in a set of two parameters, *n* and $\bar{n}$, defining the variation of the stress concentration with the density. These two parameters directly give an estimation of the evolution of K and $\bar{K}$, because of the different architectures described in the previous section.

**Figure 4:**
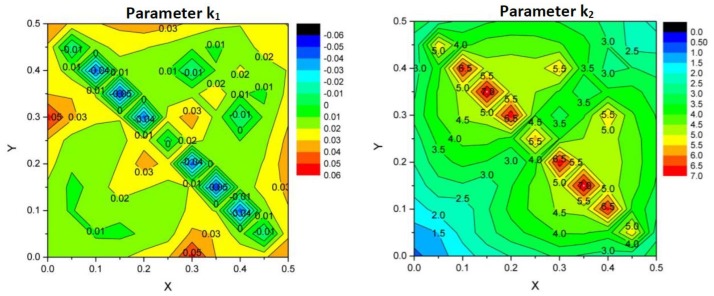
Polynomial parameters *k*_1_ and *k*_2_ for the dependence of density on the struts radii.

One must remember that the density is not set only by the value of the struts radius, but also by the architecture defined by x and y. Indeed, depending on the architecture, the connectivity and the number of struts in the representative volume are affected, and the density varies. The density follows a polynomial relation with the radius value, as follows:

(4) $$\rho =k_{1}\times r^{2}+k_{2}\times r$$

where *k*_1_ and *k*_2_ are two parameters specific to a given architecture, defined by the x and y parameters. It is reminded that the struts radius must be expressed as a fraction of the lattice parameter, and therefore, it has the same unit as the lattice parameter. For instance, if the lattice parameter is 1 mm, then the unit of *k*_1_ is mm^−2^ and the unit of *k*_2_ is mm^−1^. Figure 4 illustrates the variation of the *k*_1_ and *k*_2_ parameters with x and y. Using these two maps, it is possible to estimate the density of any structure from the value of the strut radius. Then, by introducing the value of *ρ* into Equation (3), it is possible to estimate the stress concentration factors K and $\bar{K}$. 

### 3.2. Stress Concentration Factors

**Figure 5:**
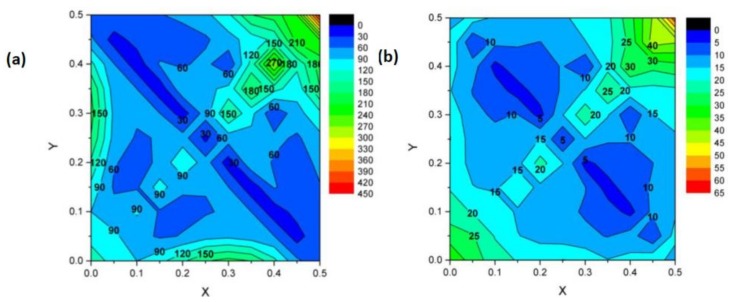
Stress concentration factor K as a function of (x, y) for two radii: (**a**) *r* = 0.05 a and (**b**) *r* = 0.1 a.

The stress concentration factor K has to be minimized to ensure a lower risk of plastic deformation and a maximal fatigue lifetime. Figure 5 illustrates the variation of K with x and y, for two strut radii. A global trend is visible. For low or high values of x + y, the stress concentration is quite high. This means that the structures similar to that of the primitive cube (x + y = 0) or to the BCC structure (x + y = 1) are prone to stress concentration. On the contrary, structures on the diagonal of the map (x + y = 0.5) exhibit a very low concentration factor K, and the mechanical strength is very promising. Figure 1 illustrates the progressive change in the architecture responsible for this trend; the increase of x + y leads to an increase in the density, and ultimately to a decrease of K.

The change in the architecture affects the density, and it leads to the variations of K on Figure 5. The decrease of K at intermediate values of x + y is mostly due to an increase of *ρ*, resulting in a decrease of the stress variations. Therefore, Figure 5 is useful to set a specific value for the K factor, for situations where the density variation is of low importance.

On the other hand, mechanical engineers are often willing to decrease the mass of parts to reduce the material consumption and the product weight. Then, *ρ* has to be minimized together with K. It becomes possible by examining the n parameter of Equation (3). It directly gives an indication of the stress concentration variation by the variation of the density for each topology.

**Figure 6:**
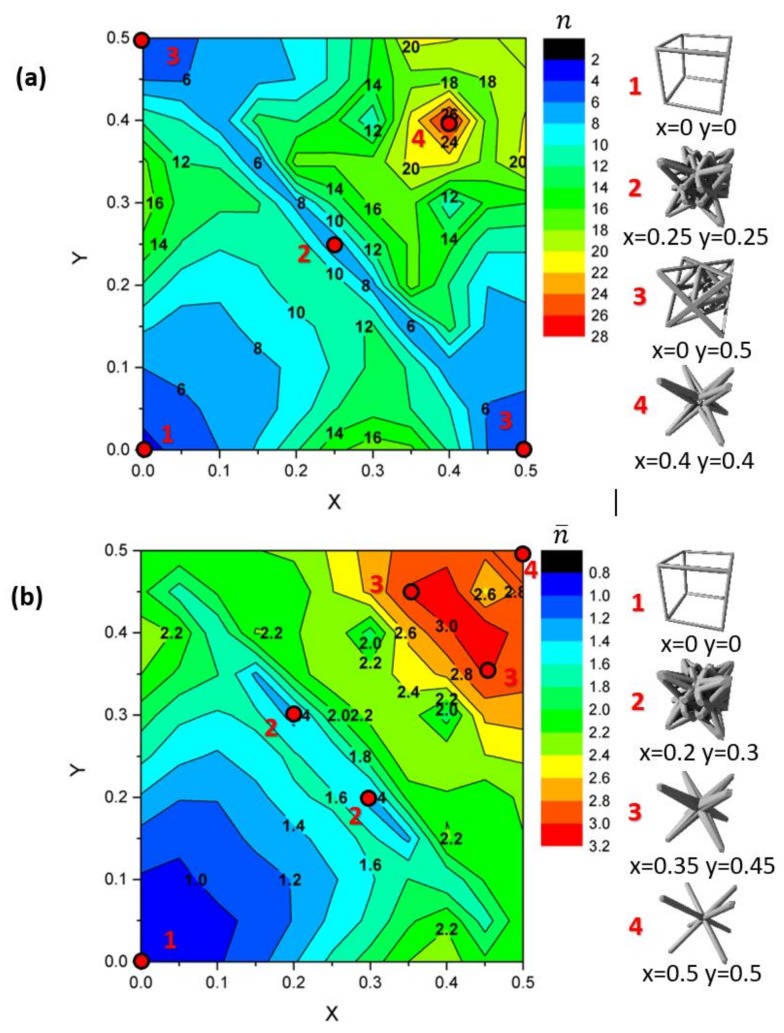
Parameter n indicating the dependence of the stress concentration factor on density for: (**a**) $K\;=\;\sigma_{m}/\tilde{\sigma }$ and (**b**) $\bar{K\;}=\;\bar{\sigma }/\tilde{\sigma }$. The optimal solutions to maximize the yield stress are highlighted in red and the solutions to minimize it are indicated in blue.

Figure 6a illustrates the variations of n parameter with the x and y parameters. The lower the n, the faster K decreases with increasing density (Equation (3)). Therefore, having low values of n is attractive for structural applications. When the density is preserved at a small value, the K factor is minimized and the risks for struts failure due to stress localization is reduced. The structures with a minimal n value would have a better resistance to localized plasticity and fatigue damage, even with decreased density. Having this in mind, it appears that the optimal structure for mechanical engineering is x = 0.0 and y = 0.0, because it minimizes the n value. The compression along the <100> direction results in pure compression stresses on the struts, and the stress concentration at the nodes remains at a very low level. The octet-truss structure (x = 0.0 and y = 0.5, FCC) and other structures located on the diagonal (x + y = 0.5) are also very interesting candidates for mass reduction because of their low n value. For a given pair of density and material, this architecture is minimizing the local stress concentration, and it should result in a higher fatigue lifetime and a higher resistance to localized damage, compared with other architectures. On the contrary, the structure x = 0.4 and y = 0.4 has the maximal n value, and belongs to the family of complex lattices with a high strut connectivity. The K factor is maximized because of the large volume fraction of the material localized at nodes where the stress is maximal. 

A similar analysis was conducted on $\bar{K}$, illustrating the evolution of the average stress concentration with the global lattice volume. Consequently, $\bar{n}$ is the sensitivity parameter of $\bar{K}$, with respect to density, and it provides information only on the global magnitude of the stress field. Using $\bar{K}$, one can discuss the occurrence of generalized plasticity or damage within an extended fraction of the part. $\bar{K}$ is especially relevant to determine the optimal structure with the maximized macroscopic yield strength, because only the average stress magnitude is considered. A low value of $\bar{n}$ means that there is great potential to preserve a low mean stress when the density is decreased. Again, the optimal architecture for a maximal yield stress at low density is x = 0.0 and y = 0.0. It allows a good compromise between the decreased density and the increased mean stress field magnitude. For a given density and a given material, this architecture results in a maximal macroscopic yield stress. The structure x = 0.2 and y = 0.3 shows a local minimum of $\bar{n}$, and it is also a suitable candidate to minimize the global stress magnitude. However, the structure with x = 0.35 and y = 0.45 maximizes the magnitude of the stress field at a given density, and the generalized plasticity is expected to occur quickly. This structure may be beneficial for some applications, for example to form the lightweight parts by plastic deformation, and it may be useful for packaging or shaping models.

## 4. Discussion

### 4.1. Case Study and Applications

Equation (3) relates three variables *n*, *ρ*, and K. To determine the optimal parameters for a workpiece, mechanical engineers must set two of these variables, and determine the third one. When (*n*, *ρ*) or (*n*, K) are set, then the architecture has already been selected, and thus it is not a variable. This corresponds to the conventional mechanical design technique, the structure is drawn into the Computer Aided Design software, and then the strut section is set to get the suitable stress. Alternatively, the stress magnitude is set in the specifications, and the strut section is determined. For a given structure, it is not possible to choose both K and *ρ*, because they are both dependent on *n*, which is a constant. 

However, there is a third way, by considering n as a variable, and setting (K, *ρ*). It is far more convenient because it becomes possible to choose any value of K and *ρ* separately, which is impossible in conventional mechanical design. Let us consider the following specification: the target of a mechanical engineer is a value of *ρ* = 0.1 and K=100. Then, using Equation (3), the n parameter must be set to 11 to fulfil this requirement. Finally, reading Figure 6a, one can deduce relevant architectures, such as x = 0.20 and y = 0.25, or x = 0.3 and y = 0.4, for instance. All of the families of the equivalent structures are on the iso-values line n = 11 on the map, and the engineer can choose deliberately the most convenient one to manufacture. Of course, if the specification implies a joint minimization of (K, *ρ*), then n has to be minimized as mentioned previously, and the optimal choice is the trivial solution x = 0.0 and y = 0.0.

**Figure 7:**
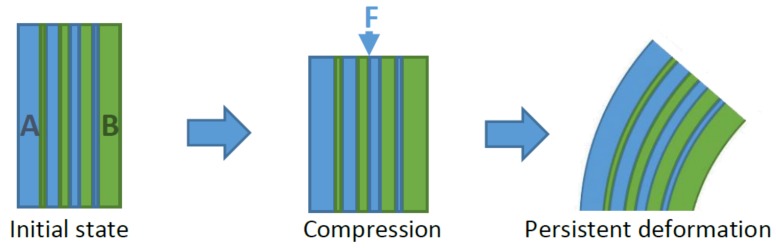
Illustration of a part composed of two different domains, A (blue, low *K*) and B (green, high *K*), with different architectures, resulting in residual stress, with F the applied force.

An example of application is the control of the deformation mode by selective plasticity. It becomes interesting to set different levels of K to control the exact area undergoing plasticity, and finally, to generate strain and stress gradients. An interesting illustration of this phenomenon is presented in Figure 7. If a bulk part is constituted of different domains called A and B, with two different lattice structures, and different *n* parameters, then the stress field will be different. Assuming domain B has a larger *K* value, it is prone to be deformed plastically at an early stage. After the plastic deformation and unloading, domain B has a larger persistent plastic strain, and it leads to the shape change of the object due to the residual stress. This unusual phenomenon is typical of functionally graded materials, and it can be tailored at will using Figure 6 to design domains A and B.

Considering now the damage, some attractive applications can be considered to control the cracking path within the domains of the specific high K value. It would be an extension of the pre-cutting usually seen on paper, but transposed to 3D volumes. When the ultimate tensile strength or the fatigue strength of the bulk is known, the optimal choice of K by adjusting n is possible, while keeping a low density in any situation.

### 4.2. Fatigue Strength

The fatigue strength has been studied for specific cases in the literature by experimental and modeling works. The most commonly investigated structures are primitive cubic and its variants [14], the diamond structure [15,16,17,18] and rhombic dodecahedron [16,19,20]. Specific attention has been paid to the rhombic dodecahedron structure, and its mechanical properties are now well known [21]. A very large set of experimental data conducted by Yavari et al. [22] and Zhang et al. [23,24] provides a large panel of S–N curves for different levels of porosities, in the case of structures made with Ti-6Al-4V by laser or electron beam melting. The experimental dataset will be compared with the first-order estimations resulting from the estimation of the stress concentration in lattices.

**Figure 8:**
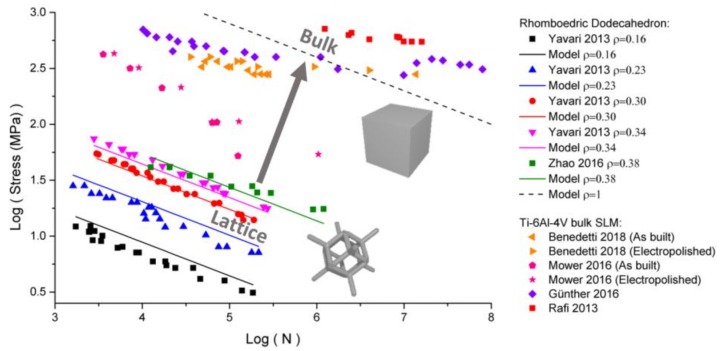
S–N curve of bulk and lattice materials (scatter plot), and the estimated values from the calculation of the stress concentration (solid lines).

The rhombic dodecahedron was generated using the symmetries of the $m\bar{3}m$ space group, as described previously. However, it cannot be produced with only two initial points, as illustrated in Figure 1. This structure requires an initial set of three points, namely: A (0, 0); B (0, 0.5), and C (0.25, 0.25). It is therefore similar to an FCC structure, having all of its tetrahedral sites filled with a node. The resulting structure is illustrated in Figure 8. The structure was generated for different struts radii corresponding to the relative densities between 0.16 and 0.38, in agreement with the authors of [22,23]. The n parameter was found to be *n* = 9.2 for this structure. Therefore, the stress concentration factor *K* is within the range of 16–49, when the relative density is in the range of 0.16–0.38.

The fatigue strength of rhombic dodecahedron is illustrated in Figure 8 [22,23]. In addition, the fatigue experimental data for the bulk Ti-6Al-4V produced by selective laser melting is superimposed in the same plot [25,26,27,28]. The S–N curves follow the Basquin equation during the high cycle fatigue, as follows:

(5) $$\Delta \sigma \cdot N^{A}=C$$

where A and C are the material parameters, Δσ is the amplitude of stress, and *N* the number of cycles to failure. The parameters A and C were identified by fitting the experimental dataset for the rhombic dodecahedron lattices and the Ti-6Al-4V alloy. 

**Figure 9:**
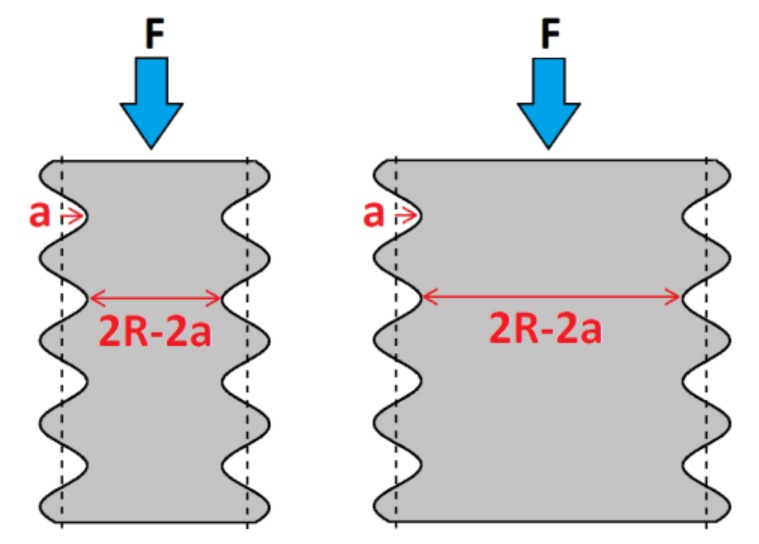
Variation of the effective beam section due to surface roughness, with F the applied force.

The decrease of density from *ρ* = 0.38 to 0.16 results in a decrease of the failure stress. This is mainly due to the stress concentration at the nodes in the lattice, resulting in an early fracture of the material compared to the bulk material. There are two levels of stress concentration. The first one is the K factor, examined previously. The second one, denoted $K_{strut}$, is as a result of the surface roughness on the struts. The size of the defects is denoted *a* in Figure 9, and results in a local strut radius R−*a*. Therefore, the local stress near a cavity increases because of the local section reduction. The concentration factor is then given by Equation (6), as follows:

(6) $$K_{strut}={\left(\frac{R}{R-a}\right)}^{2}$$

As a result, the failure stress σ is given by the following:

(7) $$\sigma =\frac{\sigma_{B}}{K\cdot K_{strut}}$$

with *σ*_B_, the stress in the bulk material for a density *ρ* = 1. In the specific case of a single cycle *N* = 1, *σ*_B_ corresponds to the parameter C in Equation (5).

According to Yavari et al. [22], the average error between the nominal struts diameter and the effective value is between 20 and 40 µm. For the rhombic dodecahedron with a lattice parameter of 1 mm, the parameters for Equation (4) are *k*_1_ = 12.51 mm^−2^ and *k*_2_ = 1.76 mm^−1^. Using Equation (4), the radius of the beams varies from 0.06 to 0.11 for a density from 0.16 to 0.38. Using an average defect size, *a* = 30 µm, the stress concentration $K_{strut}$ due to the defects is within the range of 1.8–3.6. The stress concentration K due to the architecture is obtained from Equation (3), and varies from 16 to 49 for the density range considered. Therefore, the factor K is about one order of magnitude larger than $K_{strut}$, and the effect of the architecture is predominant for the fatigue strength compared to the effect of the defects. However, the factor $K_{strut}$ cannot be neglected, because it would lead to an incorrect estimation of the S–N curves.

In Figure 8, the experimental points corresponding to lattice structures can be fitted with a slope of −0.3, corresponding to A = 0.3 for all of the values of the relative density. The experimental data for the bulk materials show a similar value of A, but with a large discrepancy due to some additional effects of the defects and processing parameters that become predominant in the bulk parts.

The vertical offset of the lattice S–N curves increases monotonously with density for the lattice structures. Therefore, the vertical offset is mainly driven by the stress concentration due to the occurrence of large open porosities in the lattices. The parameter *n* = 9.2, found by the finite element modeling, helps to set a phenomenological model to predict the S–N curves. Using C = 25 GPa for the bulk material, it becomes possible to predict with a suitable accuracy all of the data set for rhombic dodecahedron for all of the density range (solid lines in Figure 8). The change for the vertical offset is entirely explained by the stress concentration factors K and $K_{strut}$. The stress *σ_B_* follows the S–N curve for the bulk material (dashed line), and the apparent decrease of the vertical offset with decreasing density is due to the decrease of σ, caused by the stress concentration, following Equation (7).

Using extrapolation at *ρ* = 1 for the bulk material, the estimation of the bulk fatigue strength *σ_B_* is within an acceptable range between the measurement of Benedetti et al. [25] and that of Gunther et al. [26]. The estimated bulk properties are one order of magnitude higher than the data of Mower et al. [27], and one order lower than the one of Rafi et al. [28]. This illustrates the large variability of the fatigue strength, depending on the processing conditions, and the conclusions concerning the prediction of the S–N curves should be considered carefully. A significant effect of the laser scanning parameters, the powder properties, and of the possible annealing treatments may completely affect the final fatigue lifetime. The stress concentration analysis should be used as a first estimation of the possible fatigue strength so as to determine the optimal architecture. The exact quantification of the S–N curve can be obtained accurately only by an extensive experimental study with conventional fatigue tests.

To conclude, the n parameter, indicated in Figure 6, is a powerful tool to predict the local maximal stress in a lattice structure. This information can be put into the Basquin equation to produce a first-order estimation of the fatigue strength of the lattice structures.

## 5. Conclusions

Stress concentration was determined during the uniaxial compression of cubic lattice structures by finite element modeling. Maximal stress was identified by statistically analyzing the stress over the population of the elements. This method provides a reliable estimation of the maximal stress independent of the possible occurrence of singularities in the finite element calculation. The stress concentration factor K was deduced, and its variation with the relative density was determined. The parameter n is a relevant indicator of the performance of each structure to resist localized plasticity and damage during fatigue loading. The variation of the fatigue strength with density was successfully predicted in the case of the rhombic dodecahedron using the n parameter. The use of n is therefore applicable to estimate the stress concentration and the fatigue strength when the properties of the bulk material are known from the literature. From now on, the lifetime of a product can be ruled not only by the control of the density, but also by the relevant choice of the x and y architectural parameters. From these considerations, it is possible to independently adjust the weight of the part and the lifetime by adjusting the architecture within the part at will.
